# Bell’s Palsy secondary to Acute Varicella-Zoster Meningitis in an Immunocompetent 37-year-old Male.

**DOI:** 10.51894/001c.143470

**Published:** 2025-09-30

**Authors:** Cirus K. Shiran, Basil Peechakara, Maen Saleh, Alexandar Tobar

**Affiliations:** 1 College of Osteopathic Medicine Michigan State University; 2 Neurology Garden City Hospital

## 122

### INTRODUCTION

Bell’s Palsy is a unilateral facial muscle weakness caused by dysfunction of the facial nerve, which innervates muscles of facial expression bimodally. Lesions of the lower motor neurons in the corticobulbar tract (CBT) result in complete ipsilateral symptoms (peripheral lesions), while lesions of the upper motor neurons in the CBT (central lesions) cause contralateral symptoms sparing the upper forehead. Common causes include viral infections, ischemia, inflammatory disorders (Guillain-Barré syndrome, multiple sclerosis), and acute cold exposure. Early glucocorticoid treatment within 48–72 hours of symptom onset is recommended. Reactivation of VZV (Varicella-Zoster Virus) can cause Bell’s Palsy and is a known etiology of viral meningitis. Rarely, both conditions co-occur, even in immunocompetent individuals, as highlighted in this case study.

### CASE DESCRIPTION

A 37-year-old male with no significant medical history presented to a community hospital with sudden severe headache, fever, neck pain, stiffness, nausea, photophobia, and phonophobia. Initial CT and MRI scans were unremarkable. Suspecting meningitis, clinicians performed a lumbar puncture, and CSF analysis confirmed VZV. The patient was treated with acyclovir and discharged in stable condition. Four days later, he returned with persistent headache and new neurological deficits consistent with a left lower facial nerve lesion. He reported symptom onset shortly after discharge, attributed to poor pain management status post discharge. For the Bell’s Palsy, A single dose of IV methylprednisolone was administered, which has comparable if not superior efficacy to oral prednisolone. Treatment, regardless of approach, is most effective within 48–72 hours of symptom onset. Pain control and stabilization followed, and neurology prescribed muscle relaxants. Ophthalmology addressed left eye exposure keratopathy, and the patient was advised to pursue speech pathology for facial muscle rehabilitation and outpatient follow up with neurology. At discharge, he was given erythromycin ointment, artificial tears, and orphenadrine in addition to his other medications.

### CONCLUSION

Although rare, patients with VZV meningitis should be informed about potential facial nerve complications and advised to seek immediate medical attention if symptoms occur. Timely neurological exams are critical for early complication detection and treatment within the optimal window.

